# Over-Expression of the *ATP5J* Gene Correlates with Cell Migration and 5-Fluorouracil Sensitivity in Colorectal Cancer

**DOI:** 10.1371/journal.pone.0076846

**Published:** 2013-10-04

**Authors:** Hongbo Zhu, Linlin Chen, Wei Zhou, Zhongting Huang, Jingzi Hu, Sheng Dai, Xiaowei Wang, Xuefeng Huang, Chao He

**Affiliations:** 1 Department of Colorectal Surgery, Sir Run Run Shaw Hospital, School of Medicine, Zhejiang University, Hangzhou, China; 2 Key Laboratory of Biotherapy of Zhejiang province, Sir Run Run Shaw Hospital, School of Medicine, Zhejiang University, Hangzhou, China; 3 Department of Internal Medicine, Aviation Medical Evaluation and Training center of Airforce in Hangzhou, Hangzhou, China; Sun Yat-sen University Medical School, China

## Abstract

Recently we found that *ATP5J* was over-expressed in tissue samples from patients with colorectal cancer. However, the clinical significance and function of the over-expression of *ATP5J* in these patients remains unclear. We investigated these issues in the current study. Our results indicated that expression of *ATP5J* was significantly higher in colorectal cancer tissue than in adjacent tissue, and it was also significantly higher in metastatic lymph nodes than in primary cancer tissue (*P*<0.05). A correlation between *ATP5J* expression and tumor differentiation was detected, but no correlation with gender, age, T stage, lymph node metastasis, or survival status was observed. Down-regulation of *ATP5J* expression attenuated the ability of cell migration and increased the sensitivity to 5-fluorouracil (5-Fu) in cells of the DLD1 cell line. Inversely, up-regulation of *ATP5J* expression enhanced cell migration and decreased 5-Fu sensitivity, suggesting that the function of ATP5J in colorectal cancer might involve cell migration and 5-Fu sensitivity.

## Introduction

Colorectal cancer is the second most common cancer in the United States and other developed countries [1,2], and its incidence is increasing year by year in developing countries [3,4]. As we know, carcinogenesis and development of colorectal cancer comprises a series of complicated processes those involve multiple genes and steps. Although a growing body of genes have been reported in literature [5–7], the search for new genes that could be related to carcinogenesis, development, diagnosis or treatment of colorectal cancer continues. Therefore, we searched the Sage database and confirmed the gene candidates of interest by reverse transcription polymerase chain reaction (RT-PCR). Ultimately, we identified a gene, *ATP5J*, that was over-expressed in the colorectal cancer.

A component of F_0_, ATP5J is a protein located in the mitochondria that is a lipid-soluble part of ATP synthetase [8]. The precursor of ATP5J consists of 108 amino acids and it will be shorn of 32 amino acids [9]. The product containing the residual 76 amino acids is retained in the mitochondria for energy transfer [9]. Traditionally, ATP5J has been thought to recover the exchange between inorganic phosphorus and ATP as well as the activity of ATPase that is inhibited by oligomycin [10]. Recent research, however, has shown that ATP5J is extensively distributed on the surface of vascular endothelial cells [11,12]. Therefore, it may also be secreted into the blood and circulated to combine with the β subunit of ATP synthetase located on the surface of vascular endothelial cells. Subsequently, the activity of phospholipase A2 may be inhibited by the activation of the related signaling pathway, which will be followed by the inhibition of the synthesis of prostaglandin that promotes vasoconstriction [12]. Recenetly, some literatures have shown that *ATP5J* gene is overexpressed in a series of cancers including renal cell carcinoma and hepatocellular carcinoma [13,14]. However, the function of over-expressed *ATP5J* in cancers still has not been documented.

According to the introduction of THE HUMAN PROTEIN ATLAS (http://www.proteinatlas.org/ENSG00000154723/normal), ATP5J protein can be expressed in many normal tissues or organs including colon and rectum. Our preliminary data also confirmed this phenomenon. More importantly, our data demonstrated that *ATP5J* was over-expressed in the colorectal cancer compared with normal tissue. The aim of the current study was to investigate the clinical significance and function of the over-expression of *ATP5J* in colorectal cancer cells. Our results showed that *ATP5J* was over-expressed in tissue samples from patients with colorectal cancer and that there was a correlation between *ATP5J* expression and tumor differentiation. Furthermore, over-expression of *ATP5J* enhanced cell migration and induced resistance to 5-fluorouracil (5-Fu) in the colorectal cancer cells.

## Material and Methods

### Collection of fresh tissue samples

This study was approved by the Sir Run Run Shaw hospital and Zhejiang University Ethics Committee (NO.20100823). Fresh cancerous tissue samples were obtained directly from the operation specimens of 72 consecutive patients who had undergone surgical resections for primary sporadic colorectal adenocarcinomas at the Department of Colorectal Surgery, Sir Run Run Shaw Hospital, Hangzhou, Zhejiang, China, between September 2010 and March 2011. Written informed consent for tissue collection was obtained from all patients prior to their surgical procedures. None of these patients had had any preoperative chemotherapy or radiotherapy. The adjacent tissues were collected from more than 5 cm away from the cancerous tissue.

### Patients and clinical data collection

Paraffin sections of samples from a total of 79 patients with intact clinical data who were diagnosed with colorectal cancer between July 2006 and June 2007 at our hospital were collected for immunohistochemical staining. The mean follow-up of these patients was 52.6 months, and the overall survival rate was 73.4% at the last follow-up. Among these 79 cases, cancerous tissue and relative normally adjacent tissue pairs were studied in 51 cases, and tissue samples from metastatic lymph nodes were studied in 26 cases. All sections were prepared for immunohistochemistry on slides that were used for comparing *ATP5J* expression between different tissues.

### Cells and cell culture

Human colon cancer cell lines DLD1, RKO, SW620, SW480, and Colo320 were purchased from the Institute of Cell Research in Shanghai, China. The normal human fibroblast cell line (NHFB) was obtained from the Global Bioresource Center—American Type Culture Collection (Manassas, VA, USA). The cells were maintained in Dulbecco’s modified Eagle’s medium (DMEM) supplemented with 10% (v/v) heat-inactivated fetal bovine serum (FBS), 1% glutamine, and a 1× antibiotic-antimycotic mixture (Invitrogen, Beijing Office, Beijing, China). All cells were cultured at 37°C in a humidified incubator containing 5% CO_2_ and would be used for RT-PCR analysis. In addition, DLD1 as well as SW620 cells would be subsequently used for a series of other experiments.

### Reverse transcription PCR

The fresh tissue samples of 100 µg were homogenized using a grinder with liquid nitrogen followed by lysis with TRIZOL reagent (Invitrogen Beijing Office, Beijing, China). Cultured cells mentioned as above were directly lysed using TRIZOL reagent after treatment. RNA was extracted following the manufacturer’s instructions. One microgram RNA from each sample was reverse-transcribed into cDNA using random hexamers as reverse transcription primers (Applied Biosystems Shanghai Office, Shanghai, China). Then the typical polymerase chain reaction (PCR) for the *ATP5J* gene was performed. The human *GAPDH* gene was used as an internal control for normalization of the mRNA amount. The sequences of the primers were as follows: 5’-TCAGCCGTCTCAGTCCATTT-3’ and 5’-CCAAACATTTGCTTGAGCTT-3’ for *ATP5J*; 5’-ACCACAGTCCATGCCATCAC-3’ and 5’-TCCACCACCCTGTTGCTGTA-3’ for *GAPDH*.

### Immunohistochemical staining

Immunostaining was performed using a MaxVision kit (Maixin Biol, Fuzhou, China). Briefly, 4-µm-thick sections were cut from formalin-fixed, paraffin-embedded tissue blocks, mounted on polylysine coated slides, dewaxed in xylene, and rehydrated through a graded series of ethanol solutions. After deparaffinization, slides were treated with 3% hydrogen peroxide in methanol solution for 10 minutes to quench endogenous peroxidase activity, then antigen-retrieval treatment was performed at 121°C (autoclave) for 5 minutes in 10 mM sodium citrate buffer (pH 7.4). Nonspecific bindings were blocked by treating slides with 10% normal goat serum for 10 minutes. Thereafter, the slides were incubated with the ATP5J antibody (Cell Applications, San Diego, CA, 1:8000 dilution) overnight at 4°C. Next, slides were incubated with one drop of MaxVision reagent for 30 minutes at room temperature. Color development was conducted using 0.05% diaminobenzidine and 0.03% hydrogen peroxide in 50 mM Tris-HCl, pH 7.6, for 5 minutes. Finally, the slides were counterstained with 1% Meyer’s hematoxylin. As a negative control, tissue sections were treated with normal serum instead of ATP5J antibody.

### Evaluation of staining

All sections were scored blindly under a light microscope. Cells with clear structure and brown-yellow granules that were higher than the background were considered positive; otherwise, cells were considered negative. For each slide, 5–10 high-magnification fields (×400) were examined. Then the slides were scored according to the percentage of positive cells: 0 (<5%), 1 (5–25%), 2 (26–50%), 3 (51-75%), or 4 (76–100%). Simultaneously, slides were scored according to staining intensity: 1 (yellowy), 2 (brown-yellow), or 3 (brown) [15]. The final score was calculated as the sum of these two scores.

### Western blot analysis

The cells were washed with cold PBS and subjected to lysis in Laemmli’s lysis buffer. Equal amounts of lysate were separated using 10% sodium dodecyl sulfate-polyacrylamide gel electrophoresis (SDS-PAGE) and then transferred to Hybond enhanced chemiluminescence (ECL) membranes (Amersham Bioscience, England). Membranes were then blocked with PBS buffer containing 5% low-fat milk and 0.05% Tween-20 for 1 hour or overnight at 4°C, washed three times with PBS containing 0.05% Tween-20 (PBST), and incubated with ATP5J antibody for at least 1 hour at room temperature. After being washed with PBST again, membranes were incubated with peroxidase-conjugated secondary antibodies and developed with a chemiluminescence detection kit (ECL kit, Amersham Bioscience, England). Rabbit anti-human ATP5J antibody was purchased from Cell Applications. β-actin was used as a loading control.

### Construction of the plasmid expressing *ATP5J*

A pOTB7 plasmid containing the *ATP5J* sequence was purchased from Invitrogen (Clone ID 3357779). The *ATP5J* sequence was cloned into a p△E1sp1A plasmid using EcoRI/XhoI enzymes. Then it was further digested and cloned into a pcDNA3.1(+) plasmid using BamHI/HindⅢ enzymes to obtain a new plasmid named pcDNA3.1(+)/ATP5J. After the expression of *ATP5J* was confirmed, this new plasmid was used for the next part of the study.

### Cell transfection and stable colony selection

For transfection, cells were plated in 24-well plates at a density of 1x10^5^ cells per well and allowed to grow overnight to 90–95% confluency. The next day, the cells were transfected with the mixture of 0.8 µg ATP5J shRNA plasmid (Origene, USA), pcDNA3.1(+)/ATP5J plasmid, or negative control plasmid (Origene, USA) plus 2 µl Lipofectamine 2000 (Invitrogen, Carlsbad, CA) in 100 µl serum-free medium according to the manufacturer’s instructions. To produce stably transfected cells after transfection with the corresponding plasmid, 10 µg/ml of puromycin (Roche, Mannheim, Germany) was added at 48 hours to the medium (DMEM + 15% FBS). The cells were left in selective medium for 2 weeks; after this time they were trypsinized and recultured in selective medium for propagation.

### Cell viability assay

Cell viability was determined using an MTT assay (3-(4,5 dimethylthiazol-2yl)-2,5 diphenyltetrazolium bromide; Sangon, Shanghai, China). Briefly, cells (5x10^3^ per well) were seeded in 96-well plates for observation at a series of time points. Then 100 µl of MTT solution (10 mg/ml) was added to each well, and incubation was continued for 4 hours before the medium was removed. Next, 100 µl of dimethyl sulfoxide (DMSO) was applied to each well for another 10 minutes. Finally, the value of OD_570nm_ was determined; this value represents cell viability. Each experiment was performed in quadruplicate and was repeated at least twice.

### Cell clonogenic assay

1000 Cells were cultured in 10-cm dishes with normal culture medium in an incubator containing 5% CO_2_ at 37°C for 14 days. Individual colonies (>50 cells per colony) were fixed and stained with a solution containing 0.25% crystal violet stain and 20% ethanol for 20 minutes. The colonies were counted using Optimas software (Media Cybernetics LP, Silver Spring, MD, USA). Each experiment was done in triplicate and was repeated twice.

### Flow cytometry assay

Cells were trypsinized and washed once with cold PBS. Then the cells were fixed with cold 70% ethanol overnight at 4°C. Thirty minutes before they were assayed, propidium iodide (PI) staining was performed as described previously [16–18]. Flow cytometry assays were performed in the Core Lab at Sir Run Run Shaw Hospital.

### Wound-healing assay

Cell migration was studied using a scratch wound–healing assay. Cells (5x10^5^ per well) were seeded in six-well plates and allowed to adhere for 24 hours. The cells were treated with 10 µg/ml mitomycin C (Sigma-Aldrich, St. Louis, MO) for 3 hours, washed with PBS, then simply wounded with a pipette tip. Fresh full medium was added, and the cells were allowed to close the wound for 48 hours or less. Photographs of the same wound position were taken at corresponding time points. The experiment was performed in triplicate.

### Cell migration assay

Cells were trypsinized and resuspended in DMEM containing 1% FBS at a density of 1x10^6^ cells/ml. In all, 100 µl of the cell suspension was plated on a 24-well Transwell (Costar, Corning, NY). DMEM (600 µl) containing 10% FBS was placed in the lower chamber. After incubation for 24 hours, cells remaining in the upper chamber were removed carefully using a cotton swab, and the membrane was cut off using an operating knife. The side facing the lower chamber was stained with 0.05% crystal violet stain, and the attached cells were counted under a light microscope. The experiment was repeated three times.

### Statistical analysis

Correlation between *ATP5J* expression and clinical features was assessed using correlation analysis, and differences among the groups were assessed using the Wilcoxon matched-pair test or by ANOVA using SPSS15.0 software (SPSS, Inc., Chicago, USA). *P* < 0.05 was considered statistically significant.

## Results

### *ATP5J* was over-expressed in clinical colorectal cancer tissues

**Figure 1:**
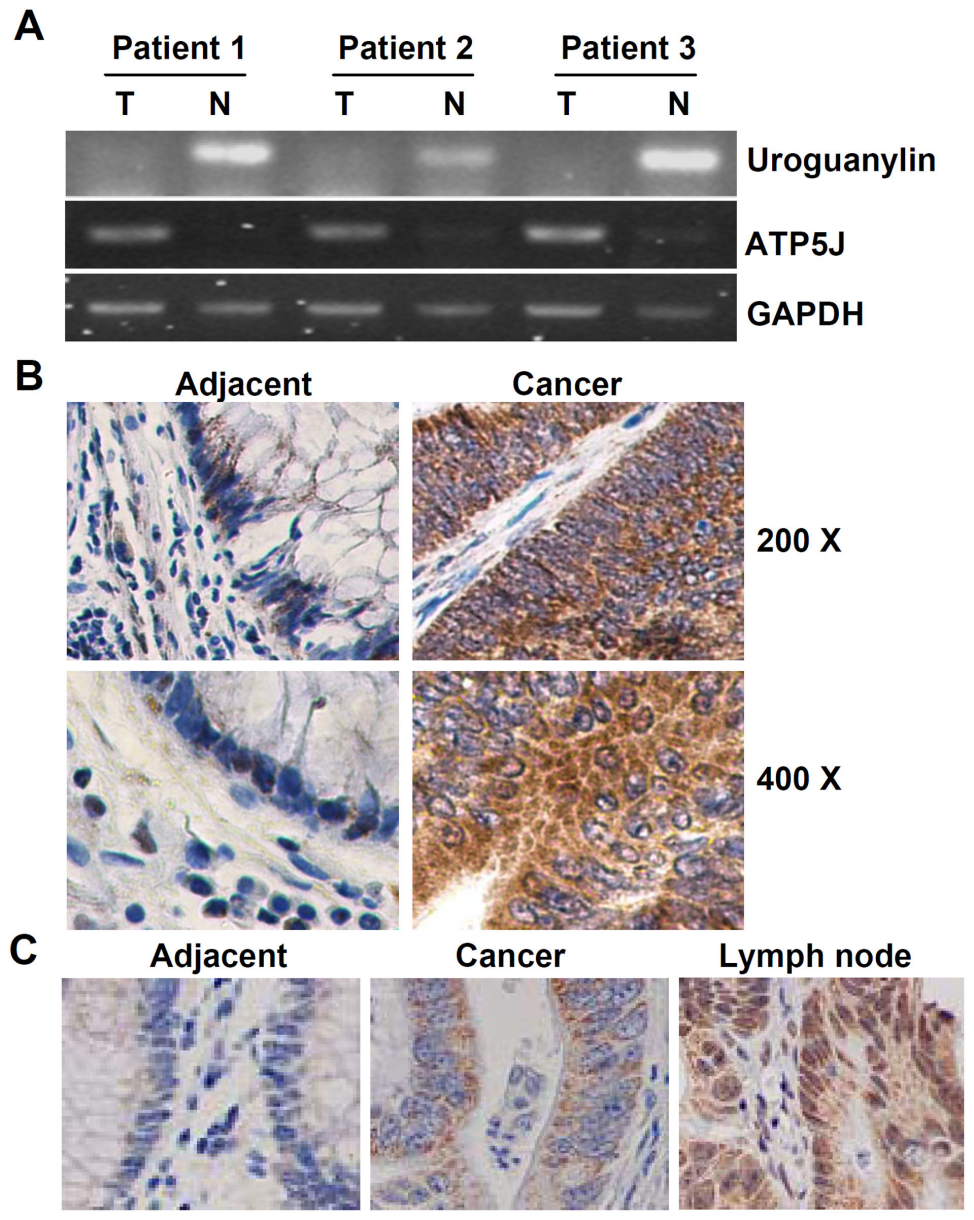
Condition of *ATP5J* expression in colorectal cancer patients. (A) RT-PCR results for the *ATP5J* and *uroguanylin* genes. (B) Immunohistochemical staining results for adjacent tissue and cancer tissue. (C) Immunohistochemical staining results for adjacent tissue, cancer tissue, and metastatic lymph node tissue.

In the Sage database (http://cgap.nci.nih.gov/SAGE/AnatomicViewer), we searched for a range of candidate genes those were differentially expressed in colorectal cancer. Several interesting genes were identified, including tetraspanin TM4-C, oligophrenin 1, ATP5J, transmembrane glycoprotein A33 precursor, DHRS9, cytidine deaminase, uroguanylin, and dual-specificity phosphatase 1. However, our primary results from RT-PCR indicated that only uroguanylin was reduced and that *ATP5J* was over-expressed in colorectal cancer (Figure 1A). The other genes were unchanged or undetectable in our experiment (data not shown). Because expression of uroguanylin and its function in colon cancer has been clarified in a previous report [19], but not for the *ATP5J* gene, the latter was chosen as the unique objective in our study.

To confirm the expression of *ATP5J* in colorectal cancer, we further analyzed the expression of *ATP5J* mRNA by RT-PCR in fresh tumor tissues and adjacent normal tissues from the 72 consecutive patients (Table 1). The results indicated that positive percentages of RT-PCR for *ATP5J* in tissues from colorectal cancer patients reached approximately 54.17%, which was similar between colon cancer and rectal cancer. However, expression of *ATP5J* was significantly higher in colorectal cancer tissue than in normal tissue among the PCR-positive patients (Table 1, *P*<0.01). Our immunohistochemical staining results from the paraffin sections of another 51 patients mentioned in *Patients and clinical data collection* section indicated a similar result, but with a higher positive ratio (Figure 1B and Table 1, *P*<0.05). Further immunostaining results also demonstrated that *ATP5J* expression was significantly higher in metastatic lymph nodes than in primary cancer tissue (Figure 1C and Table 2, *P*<0.05).

**Table 1:** Condition of ATP5J expression in colorectal tumor and adjacent tissue.

ATP5J condition	Case NO.	Positive cases	Positive cases’ distribution by ATP5J expression in tumor vs. adjacent tissue			*P* value
			T^*^ > A^*^	T = A	T < A	
mRNA by PCR	72	39 (54.2%)	32 (82.1%)	6 (15.4%)	1 (2.6%)	<0.01
Protein by IM*	51	49 (96.1%)	32 (65.3%)	15 (30.6%)	2 (4.1%)	<0.05

* T: tumor; A: adjacent; IM: immunohistochemical staining

**Table 2:** Condition of ATP5J expression in colorectal tumor and metastatic lymph nodes.

ATP5J in MLN*	Case NO.	MLN>T	MLN=T	MLN<T	*P* value
Positive	25 (96.2%)	12 (46.2%)	8 (30.8%)	5 (19.2%)	<0.05
Negative	1 (3.8%)	－	－	1 (3.8%)	

* MLN: metastatic lymph nodes

### Correlation between over-expression of *ATP5J* and clinical features of patients with colorectal cancer

To analyze the relationship between over-expression of ATP5J and clinical pathological characteristics of colorectal cancer patients, paraffin sections from 79 patients, as mentioned in section of *Patients and clinical data collection*, were collected. Immunohistochemical staining was performed on these slides and the scores were calculated as mentioned above. According to these scores, *ATP5J* expression could be further divided into two ranks: weak (≤4) and strong (>4). Then correlation analysis was done using SPSS software; the results were shown in Table 3. Among the listed clinical features, only degree of differentiation had a correlation with *ATP5J* expression. Gender, age, T stage, lymph node metastasis, as well as survival status did not show a correlation with *ATP5J* expression. These results indicated that *ATP5J* expression in well-differentiated tumors was weaker than that in poorly or moderately differentiated tumors (*P* <0.05)

**Table 3:** Correlation between ATP5J expression and patients’clinical features.

Variable	Case NO.	Weak	Strong	P Vaule
Gender				
Female	35	8	27	0.281
Male	44	15	29	
Age				
>65 yr	28	6	22	0.271
≤65	51	17	34	
T stage				
T1,2	19	6	13	0.789
T3,4	60	17	43	
Differentiation				
Well	41	16	25	0.045
Poor/moderate	38	7	31	
Histologic LNM*				
Absent	36	12	24	0.408
Present	43	11	32	
Survival status				
Survival	58	18	40	0.538
Died	21	5	16	

* LNM: lymph node metastases

### Detection of *ATP5J* expression in colon cancer cell lines

**Figure 2:**
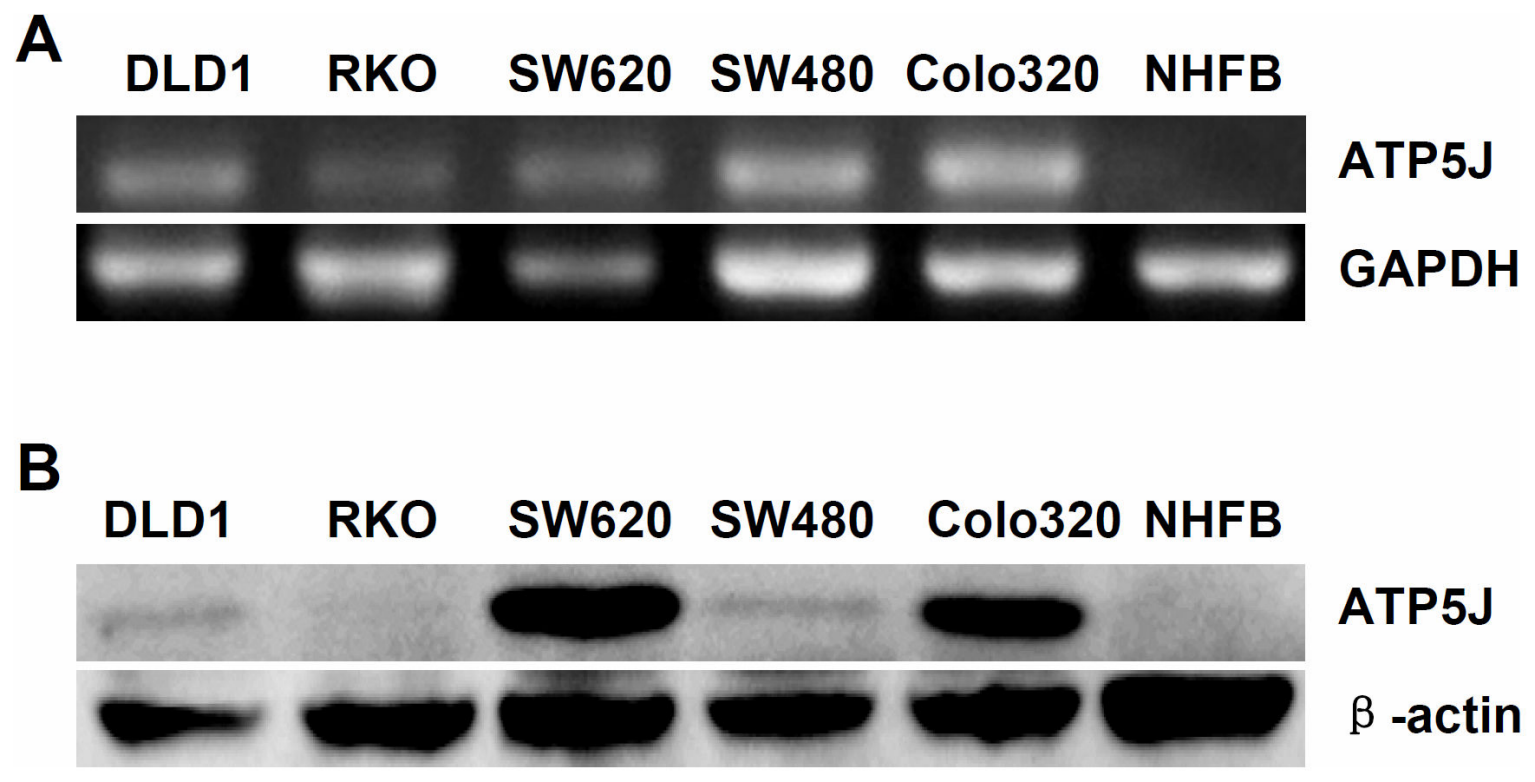
*ATP5J* is over-expressed in colon cancer cells. (A) RT-PCR results for ATP5J mRNA. (B) Western blotting results for ATP5J protein.

It was difficult to clarify the underlying function of ATP5J in colorectal cancer depending only on the clinical analyses. More molecular biological studies were needed for this purpose. Therefore, our attention was shifted from colorectal cancer patients to cell lines. To understand the expression of *ATP5J* in colorectal cancer cell lines, five colorectal cancer cell lines were collected (DLD1, RKO, SW620, SW480, and Colo320), and the normal human fibroblast (NHFB) was used as normal cell control. First, we collected the mRNA product from these cell lines and performed RT-PCR as described previously. These results showed that ATP5J mRNA was over-expressed in all of colon cancer cell lines but not in NHFB (Figure 2A). We then performed Western blotting on the protein samples of these cell lines. The results demonstrated a similar phenomenon. ATP5J protein was over-expressed in four colon cancer cell lines (except RKO) compared with NHFB (Figure 2B). Based on these results, we mainly chose DLD1 cells for the next part of our study; DLD1 showed a moderate degree of *ATP5J* expression, which was convenient for observing the down-regulation as well as the up-regulation of the gene expression in the same cell line using molecular techniques.

### Down-regulation of *ATP5J* expression attenuated cell migration and increased 5-Fu sensitivity in DLD1 cells

**Figure 3:**
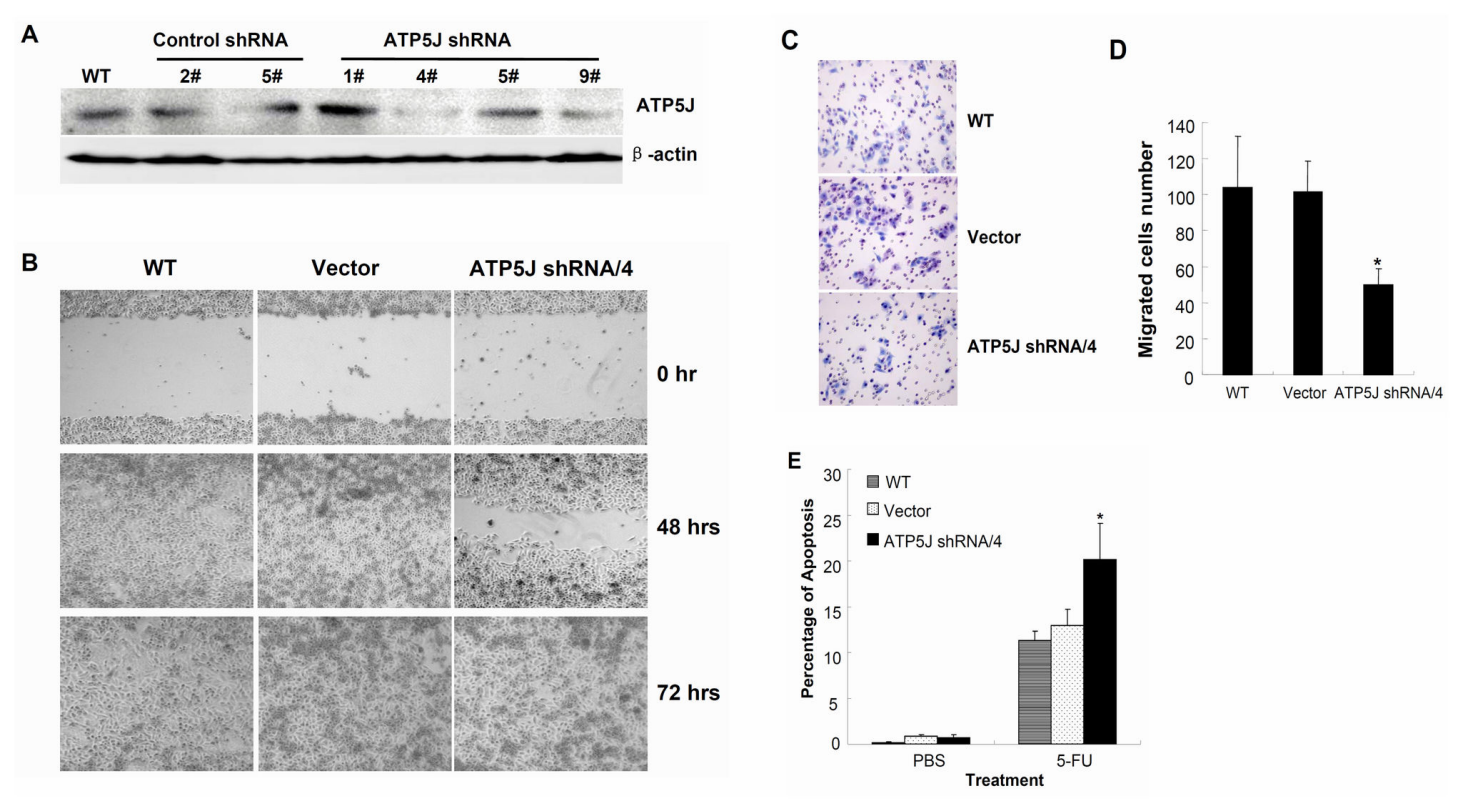
Down-regulation of *ATP5J* expression attenuated cell migration and increased 5-Fu sensitivity in DLD1 cells. (A) Western blot result for ATP5J protein in different clones of DLD1 cell after stable transfection with the same ATP5J shRNA plasmid. (B) Wound-healing assay for WT, Vector, and ATP5J shRNA/4 DLD1 cells. Data represent one of three similar experiments (original magnification: x100). (C) Migration of WT, Vector, and ATP5J shRNA/4 DLD1 cells was assayed using a 24-Transwell system. The pictures of migrated cells were taken 24 hours after seeding (original magnification: x100). Data represent one of three similar experiments. (D) Quantitative analyses of three cell migration assays. Values represent means ± SD. * *P*<0.05; (E) Apoptotic ratios of WT, Vector, and ATP5J shRNA/4 DLD1 cells after treatment with 50 µmol/L 5-Fu for 3 days. Data represent one of two similar experiments. Values represent means ± SD. *P<0.05.

First, we down-regulated *ATP5J* expression in DLD1 cells through stable transfection with a plasmid expressing shRNA targeting ATP5J. After colony selection, Western blotting was performed (Figure 3A). The expression of ATP5J was down-regulated dramatically in clone 4 and marginally in clone 9. We chose clone 4 for further study and defined it as ATP5J shRNA/4. Meanwhile, clone 2 from control shRNA was used as vector control (Vector), and wild-type DLD1 (WT) was used as a mock control.

The results of the MTT assay, cell cycle by flow cytometry, or clone formation by clonogenic assay revealed no differences in cell survival among WT, Vector, and ATP5J shRNA/4 groups (data not shown). However, data from the wound-healing assay indicated that cells in the ATP5J shRNA/4 group took >48 hours to close a scratch wound, whereas cells in the WT and Vector groups took <48 hours to heal a wound of similar size (Figure 3B). This result suggested that down-regulation of *ATP5J* attenuated the ability of cell migration in DLD1 cells. To quantify the effect of *ATP5J* down-regulation on cell migration, a further migration assay was performed. The results of this additional assay showed that the number of migrated cells in the ATP5J shRNA/4 group was significantly reduced compared with both the WT and Vector groups (Figure 3C and 3D, *P*<0.05).

In addition, we wanted to evaluate the response of cancer cells to anti-cancer therapy after the ATP5J expression was changed. For this purpose, we chose 5-fluorouracil (5-Fu) for next study, because it is a frequently used anti-metabolite chemotherapeutic drug in colorectal cancer. Our results indicated that the apoptotic ratio of ATP5J shRNA/4 group was higher than that of both the WT and Vector groups after treatment with 50 µmol/L 5-Fu for 3 days (Figure 3E, P<0.05). This result suggested that down-regulation of *ATP5J* increased the sensitivity of DLD1 cells to 5-Fu.

**Figure 4:**
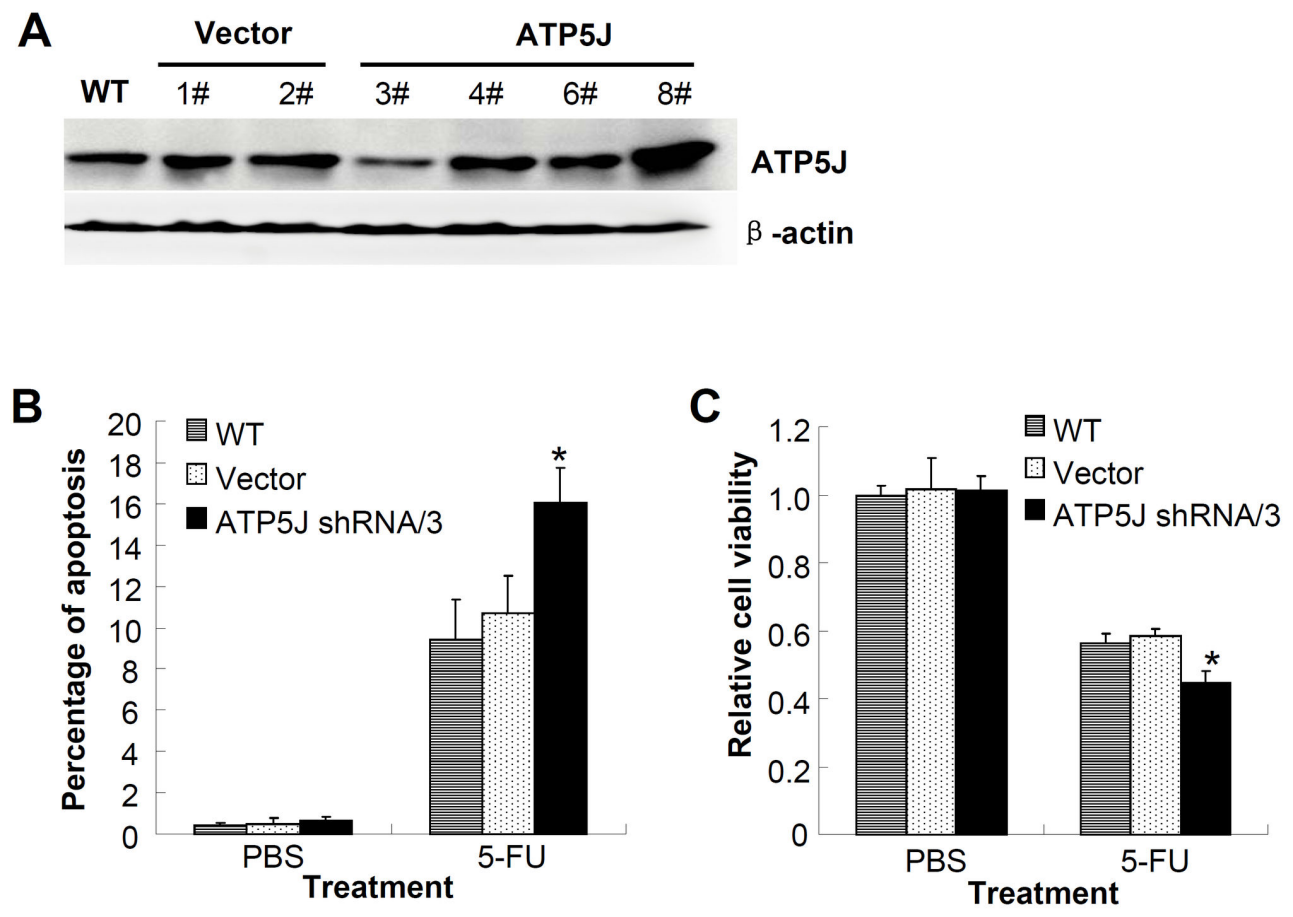
SW620 cells were more sensitive to 5-Fu after knockdown of *ATP5J* expression. (A) Western blot result for ATP5J protein in different clones of SW620 cell after stable transfection with the same ATP5J shRNA plasmid. (B) Apoptotic ratio of WT, Vector, and ATP5J shRNA/3 SW620 cells after treatment with 50 µmol/L 5-Fu for 3 days. (C) Cell viability assays of WT, Vector, and ATP5J shRNA/3 SW620 cells after treatment with 50 µmol/L 5-Fu for 3 days. Data represent one of two similar experiments. Values represent means ± SD. *P<0.05.

To further confirm the function of ATP5J in the other cell line, we knocked down the *ATP5J* expression in SW620 cells using the same plasmid as mentioned above. After colony selection, we identified one clone in which *ATP5J* expression was dramatically down-regulated; we named it ATP5J shRNA/3 (Figure 4A). The results from both the apoptosis detection and cell viability assays suggested that the ATP5J shRNA/3 group derived from SW620 cells was more sensitive to 5-Fu compared with the WT and Vector groups after treatment with 50 µmol/L 5-Fu for 3 days (Figure 4B and 4C, *P*<0.05). This result is consistent with our previous data from DLD1 cells and further validates the function of ATP5J in colon cancer cells.

### Up-regulation of *ATP5J* expression enhanced cell migration and decreased 5-Fu sensitivity in DLD1 cells

**Figure 5:**
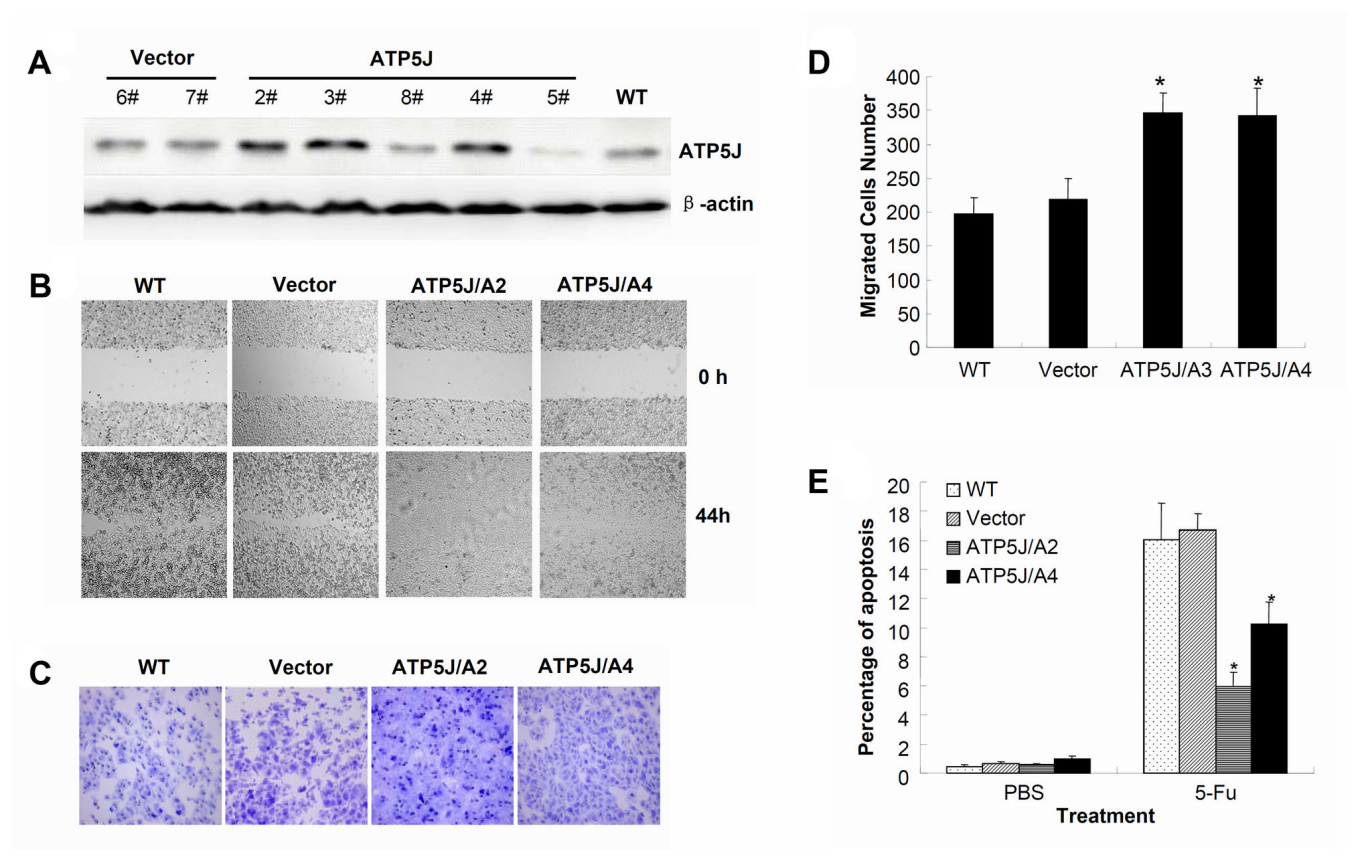
Up-regulation of *ATP5J* expression enhanced cell migration and decreased 5-Fu sensitivity in DLD1 cells. (A) Western blot result for ATP5J protein in DLD1 cells after stable transfection with pcDNA3.1(+)/ATP5J plasmid. (B) Wound-healing assay for WT, Vector, ATP5J/A2, and ATP5J/A4 cells. Data represent one of three similar experiments (original magnification: x100). (C) Migrations of WT, Vector, ATP5J/A2, and ATP5J/A4 cells were assayed using a 24-Transwell system. The pictures of migrated cells were taken 24 hours after seeding (original magnification: x100). Data represent one of three similar experiments. (D) Quantitative analysis of three cell-migration assays. Values represent means ± SD. *P<0.05. (E) Apoptotic ratios of WT, Vector, ATP5J/A2, and ATP5J/A4 cells after treatment with 50 µmol/L 5-Fu for 3 days. Data represent one of two similar experiments. Values represent means ± SD and *P<0.05.

Next, we wanted to confirm our result using an opposite condition in which *ATP5J* expression was further up-regulated. To achieve this aim, the pcDNA3.1(+)/ATP5J plasmid was stably transfected into DLD1 cells. After colony selection, Western blotting was performed (Figure 5A). The ATP5J expression was up-regulated dramatically in clones 2, 3, and 4. We randomly chose clones 2 and 4 for further study and defined them as ATP5J/A2 and ATP5J/A4, respectively. Meanwhile, clone 7 from the control plasmid was used as vector control (Vector) and the wild-type DLD1 cells (WT) were used as a mock control.

The data regarding cell survival, cell cycle, and clone formation yielded results similar to those presented above. No difference was observed among WT, Vector, ATP5J/A2, and ATP5J/A4 groups (data not shown). The wound–healing assay showed that cells in the ATP5J/A2 and ATP5J/A4 groups took <44 hours to close a scratch wound, whereas cells in the WT and Vector groups took >44 hours to heal a wound of similar size (Figure 5B). This result suggested that up-regulation of *ATP5J* enhanced cell migration in DLD1 cells. A further cell-migration assay demonstrated that the number of migrated cells in the ATP5J/A2 and ATP5J/A4 groups was significantly increased compared with those in the WT and Vector groups (Figure 5C and 5D, *P*<0.05). Meanwhile, after treatment with 50 µmol/L 5-Fu for 3 days, the apoptotic ratios of the ATP5J/A2 and ATP5J/A4 groups was significantly lower than those of the WT and Vector groups (Figure 5E, P<0.05), indicating that up-regulation of *ATP5J* induced DLD1 cells to resist to 5-Fu.

## Discussion

Our study investigated the status of *ATP5J* expression in colorectal cancer patients, and our results demonstrated that *ATP5J* was over-expressed in these patients. This result was opposite to that in Sage database. It could be due to the difference of sample size as well as sample selection bias. According to our data, there were several different conditions of ATP5J expression in colorectal cancer, one of them was reduced. In addition, the sample size used by database was very small. So it could be understood that we had a different conclusion about ATP5J expression in colorectal cancer. Moreover, our results were obtained at both mRNA and protein levels and should, therefore, be convincing.

Further results showed that the degree of differentiation was correlated with *ATP5J* expression. According to the published literatures, tumor differentiation was a prognosis factor for the patients with colorectal cancers [20,21]. Based on this, *ATP5J* expression might be correlated with the survival status, however our data did not showed this correlation. Additional, our results also indicated that *ATP5J* expression was higher in metastatic lymph nodes compared with corresponding primary cancer tissue, but it had no correlation with lymph node metastasis. The discrepancy might be caused by too many uncontrollable factors clinically, rather than by the conditions in the molecular biological studies, which were controlled exactly. It might be also caused by small size of included patients and need an investigation with large samples. Regardless of this disagreement, our further results perspicuously indicated that down-regulation of *ATP5J* expression attenuated cell migration and increased 5-Fu sensitivity in DLD1 cells. Inversely, up-regulation of *ATP5J* expression enhanced cell migration and decreased 5-Fu sensitivity, suggesting that the function of ATP5J in colorectal cancer could be involved in cell migration and 5-Fu sensitivity. As we know, 5-Fu-based adjuvant chemotherapy has been shown to be highly efficacious in reducing mortality for the node-positive colorectal cancer and has become the standard of care [1]. Our results showed the level of ATP5J could effect the sensitivity of colorectal cancer cells towards 5-Fu, which might be pointing out a potential way to enhance the anti-tumor effect of 5-Fu on the colorectal cancer.

ATP5J is a component of F_0_ that is a lipid-soluble part of ATP synthetase. It is responsible for the connection of F_0_ and F_1_ that is a water-soluble part of ATP synthetase [8]. Until now, it has been reported that some subunits of ATP synthetase are abnormally expressed in cancer cells, such as reduced quantities of β subunits or increased MDR1 expression that might be correlated with the sensitivity of cancer cells to chemotherapy or radiotherapy [22–24]. However, there are only a few of literatures showing that *ATP5J* gene is overexpressed in a series of cancers [13,14]. So far, the underlying mechanism of *ATP5J* function in colorectal cancer remains unclear and requires further investigation. Our current study might be achieving a part of the truth.

In summary, we have confirmed that *ATP5J* is over-expressed in colorectal cancer cells, and we have demonstrated a correlation between *ATP5J* expression and tumor differentiation. Moreover, over-expression of *ATP5J* might enhance cell migration and induce resistance to 5-Fu in DLD1 cells, indicating that *ATP5J* might be involved in cell migration and 5-Fu sensitivity in colorectal cancer.

## References

[1] Chau I, Cunningham D (2002) Chemotherapy in colorectal cancer: new options and new challenges. Br Med Bull 64: 159-180. doi:10.1093/bmb/64.1.159. PubMed: 12421732. 12421732

[2] Siegel R, Desantis C, Virgo K, Stein K, Mariotto A et al. (2012) Cancer treatment and survivorship statistics, 2012. CA Cancer J Clin 62: 220-241. doi:10.3322/caac.21149. PubMed: 22700443. 22700443

[3] Pathy S, Lambert R, Sauvaget C, Sankaranarayanan R (2012) The incidence and survival rates of colorectal cancer in India remain low compared with rising rates in East Asia. Dis Colon Rectum 55: 900-906. doi:10.1097/DCR.0b013e31825afc4e. PubMed: 22810477. 22810477

[4] Rehman MU, Buttar QM, Khawaja MI, Khawaja MR (2009) An impending cancer crisis in developing countries: are we ready for the challenge? Asian Pac J Cancer Prev 10: 719-720. PubMed: 19827904. 19827904

[5] Liao X, Morikawa T, Lochhead P, Imamura Y, Kuchiba A et al. (2012) Prognostic role of PIK3CA mutation in colorectal cancer: cohort study and literature review. Clin Cancer Res 18: 2257-2268. doi:10.1158/1078-0432.CCR-11-2410. PubMed: 22357840. 22357840 PMC3628835

[6] Reddavide R, Misciagna G, Caruso MG, Notarnicola M, Armentano R et al. (2011) Tissue expression of glycated apolipoprotein B in colorectal adenoma and cancer. Anticancer Res 31: 555-559. PubMed: 21378338. 21378338

[7] Watson AJ, Collins PD (2011) Colon cancer: a civilization disorder. Dig Dis 29: 222-228. doi:10.1159/000323926. PubMed: 21734388. 21734388

[8] Collinson IR, van Raaij MJ, Runswick MJ, Fearnley IM, Skehel JM et al. (1994) ATP synthase from bovine heart mitochondria. In vitro assembly of a stalk complex in the presence of F1-ATPase and in its absence. J Mol Biol 242: 408-421. doi:10.1006/jmbi.1994.1591. PubMed: 7932700. 7932700

[9] Chang JK, Scruggs P, Yang J, Ouyang M, Duetzmann A et al. (2003) Total synthesis of human and rat coupling factor-6 amide and pressor effects in the rat. Regul Pept 113: 63-69. doi:10.1016/S0167-0115(02)00303-8. PubMed: 12686462. 12686462

[10] Joshi S, Pringle MJ (1989) ATP synthase complex from bovine heart mitochondria. Passive H+ conduction through mitochondrial coupling factor 6-depleted F0 complexes. J Biol Chem 264: 15548-15551. PubMed: 2527853. 2527853

[11] Watts SW (2005) Vasoconstriction caused by the ATP synthase subunit-coupling factor 6: a new function for a historical enzyme. Hypertension 46: 1100-1102. doi:10.1161/01.HYP.0000186476.62399.6c. PubMed: 16230522. 16230522

[12] Osanai T, Magota K, Tanaka M, Shimada M, Murakami R et al. (2005) Intracellular signaling for vasoconstrictor coupling factor 6: novel function of beta-subunit of ATP synthase as receptor. Hypertension 46: 1140-1146. doi:10.1161/01.HYP.0000186483.86750.85. PubMed: 16230521. 16230521

[13] Bjerregaard H, Pedersen S, Kristensen SR, Marcussen N (2011) Reference genes for gene expression analysis by real-time reverse transcription polymerase chain reaction of renal cell carcinoma. Diagn Mol Pathol 20: 212-217. doi:10.1097/PDM.0b013e318212e0a9. PubMed: 22089348. 22089348

[14] Yang W, Lu Y, Xu Y, Xu L, Zheng W et al. (2012) Estrogen represses hepatocellular carcinoma (HCC) growth via inhibiting alternative activation of tumor-associated macrophages (TAMs). J Biol Chem 287: 40140-40149. doi:10.1074/jbc.M112.348763. PubMed: 22908233. 22908233 PMC3504728

[15] Zhu H, Zhu Y, Hu J, Hu W, Liao Y et al. (2007) Adenovirus-mediated small hairpin RNA targeting Bcl-XL as therapy for colon cancer. Int J Cancer 121: 1366-1372. doi:10.1002/ijc.22856. PubMed: 17534896. 17534896

[16] Zhang L, Gu J, Lin T, Huang X, Roth JA et al. (2002) Mechanisms involved in development of resistance to adenovirus-mediated proapoptotic gene therapy in DLD1 human colon cancer cell line. Gene Ther 9: 1262-1270. doi:10.1038/sj.gt.3301797. PubMed: 12215894. 12215894

[17] Huang X, Lin T, Gu J, Zhang L, Roth JA et al. (2002) Combined TRAIL and Bax gene therapy prolonged survival in mice with ovarian cancer xenograft. Gene Ther 9: 1379-1386. doi:10.1038/sj.gt.3301810. PubMed: 12365003. 12365003

[18] Gu J, Zhang L, Huang X, Lin T, Yin M et al. (2002) A novel single tetracycline-regulative adenoviral vector for tumor-specific Bax gene expression and cell killing in vitro and in vivo. Oncogene 21: 4757-4764. doi:10.1038/sj.onc.1205582. PubMed: 12101414. 12101414

[19] Shailubhai K, Yu HH, Karunanandaa K, Wang JY, Eber SL et al. (2000) Uroguanylin treatment suppresses polyp formation in the Apc(Min/+) mouse and induces apoptosis in human colon adenocarcinoma cells via cyclic GMP. Cancer Res 60: 5151-5157. PubMed: 11016642. 11016642

[20] Ju JH, Chang SC, Wang HS, Yang SH, Jiang JK et al. (2007) Changes in disease pattern and treatment outcome of colorectal cancer: a review of 5,474 cases in 20 years. Int J Colorectal Dis 22: 855-862. doi:10.1007/s00384-007-0293-z. PubMed: 17390145. 17390145

[21] André T, Sargent D, Tabernero J, O’Connell M, Buyse M et al. (2006) Current issues in adjuvant treatment of stage II colon cancer. Ann Surg Oncol 13: 887-898. doi:10.1245/ASO.2006.07.003. PubMed: 16614880. 16614880

[22] Zhou J, Liu M, Aneja R, Chandra R, Lage H et al. (2006) Reversal of P-glycoprotein-mediated multidrug resistance in cancer cells by the c-Jun NH2-terminal kinase. Cancer Res 66: 445-452. doi:10.1158/0008-5472.CAN-05-1779. PubMed: 16397260. 16397260

[23] Cuezva JM, Krajewska M, de Heredia ML, Krajewski S, Santamaría G et al. (2002) The bioenergetic signature of cancer: a marker of tumor progression. Cancer Res 62: 6674-6681. PubMed: 12438266. 12438266

[24] Cuezva JM, Chen G, Alonso AM, Isidoro A, Misek DE et al. (2004) The bioenergetic signature of lung adenocarcinomas is a molecular marker of cancer diagnosis and prognosis. Carcinogenesis 25: 1157-1163. doi:10.1093/carcin/bgh113. PubMed: 14963017. 14963017

